# Unique spectra of deafness-associated mutations in Mongolians provide insights into the genetic relationships among Eurasian populations

**DOI:** 10.1371/journal.pone.0209797

**Published:** 2018-12-21

**Authors:** Jargalkhuu Erdenechuluun, Yin-Hung Lin, Khongorzul Ganbat, Delgermaa Bataakhuu, Zaya Makhbal, Cheng-Yu Tsai, Yi-Hsin Lin, Yen-Hui Chan, Chuan-Jen Hsu, Wei-Chung Hsu, Pei-Lung Chen, Chen-Chi Wu

**Affiliations:** 1 Department of Otolaryngology, Mongolian National University of Medical Sciences, Ulaanbaatar, Mongolia; 2 The EMJJ Otolaryngology Hospital, Ulaanbaatar, Mongolia; 3 Department of Otolaryngology, National Taiwan University Hospital, Taipei, Taiwan; 4 Graduate Institute of Medical Genomics and Proteomics, National Taiwan University College of Medicine, Taipei, Taiwan; 5 Department of Otolaryngology, National Center for Maternal and Child Health, Ulaanbaatar, Mongolia; 6 Department of Medical Genetics, National Taiwan University Hospital, Taipei, Taiwan; German Cancer Research Center (DKFZ), GERMANY

## Abstract

Genetic factors are an important cause of idiopathic sensorineural hearing impairment (SNHI). From the epidemiological perspective, mutations of three deafness genes: *GJB2*, *SLC26A4*, and *MT-RNR1*, are much more prevalent than those of other genes worldwide. However, mutation spectra of common deafness genes differ remarkably across different populations. Here, we performed comprehensive genetic examination and haplotype analyses in 188 unrelated Mongolian families with idiopathic SNHI, and compared their mutation spectra and haplotypes to those of other European and Asian cohorts. We confirmed genetic diagnoses in 18 (9.6%) of the 188 families, including 13 with bi-allelic *GJB2* mutations, three with bi-allelic *SLC26A4* mutations, and two with homoplasmic *MT-RNR1* m.1555A>G mutation. Moreover, mono-allelic mutations were identified in 17 families (9.0%), including 14 with mono-allelic *GJB2* mutations and three with mono-allelic *SLC26A4* mutations. Interestingly, three *GJB2* mutations prevalent in other populations, including c.35delG in Caucasians, c.235delC in East Asians, and c.-23+1G>A in Southwest and South Asians, were simultaneously detected in Mongolian patients. Haplotype analyses further confirmed founder effects for each of the three mutations, indicating that each mutation derived from its ancestral origin independently. By demonstrating the unique spectra of deafness-associated mutations, our findings may have important clinical and scientific implications for refining the molecular diagnostics of SNHI in Mongolian patients, and for elucidating the genetic relationships among Eurasian populations.

## Introduction

Hearing impairment is the most common inherited sensory defect. It is estimated that permanent sensorineural hearing impairment (SNHI) occurs in approximately 1.9 per 1000 live births [1], and with late-onset SNHI included, the disorder may affect 2% of school-age children [2, 3]. More than 50% of SNHI cases in children are attributed to genetic causes, and are therefore classified as hereditary hearing impairment (HHI) [4]. To date, more than 100 genes have been identified as causally related to HHI (http://hereditaryhearingloss.org).

Among the plethora of HHI genes, mutations in three: *GJB2* (MIM *121011), *SLC26A4* (MIM *605646), and the mitochondrial 12S rRNA gene (*MT-RNR1*; MIM *561000), are particularly prevalent in deaf patients across different populations [4]. Predominant mutations in these genes differ significantly across populations. For instance, c.35delG, c.167delT, and c.235delC are the most common *GJB2* mutations in Caucasians [5–8], Ashkenazi Jews [9], and East Asians [10–12], respectively; whereas the *GJB2* c.-23+1G>A mutation was identified uniquely in Southwest [13, 14] and South Asians [15, 16]. Similarly, predominant *SLC26A4* mutations differ among populations, including p.T416P and c.1001G>A in Caucasians [17, 18], p.H723R in Japanese [19] and Koreans [20], and c.919-2A>G in Han Taiwanese [21] and Han Chinese [22]. These findings underscore the indispensability of collecting regional data when genetic examination for SNHI is performed in a specific population.

The genetics of SNHI in the Mongolian population have been documented in several previous studies [23–26]. However, most of these studies were conducted in cohorts recruited from the Inner Mongolia region of China; only limited numbers of Mongolian patients were included in these studies, and the admixture of other ethnic populations could not be excluded because of inter-population marriage [23–25]. Hearing-impaired patients from Mongolia have been the subject of only one previous study [26]. However, this study focused on *GJB2* mutations, and the contribution of other deafness genes to SNHI in these patients was not addressed [26].

The scientific value of investigating genetic diseases in Mongolian patients also lies in the geographic location of Mongolia. As an intersection between the European, Middle Eastern, and East Asian civilizations, dissecting the genetic underpinnings of Mongolians may offer insights into the genetic diversity and genetic relationships among the Eurasian populations [27–30]. In this study, we performed comprehensive mutation screening of three common HHI genes in a large cohort of Mongolian patients, and then conducted haplotype analyses to decipher the origins of SNHI-related mutations with reference to other European and Asian populations.

## Methods

### Subjects

From November 2016 to January 2018, a total of 188 unrelated Mongolian families with idiopathic bilateral SNHI were recruited from the EMJJ Otolaryngology Hospital and the Department of Otolaryngology, National Center for Maternal and Child Health, Ulaanbaatar, Mongolia. Patients were excluded if they (1) were aged more than 40 years, (2) had conductive or mixed-type hearing impairment, (3) had previous noise or ototoxic medical exposure, (4) had a history of perinatal insults, such as prematurity or kernicterus, or (5) had no complete records of their medical history available.

For the proband of each family, comprehensive family history, personal medical history, physical examination, audiological results, and imaging results were ascertained. The audiological results were evaluated with pure tone audiograms or auditory brainstem response, depending on age or neurological status [31]. For imaging studies, non-contrast temporal bone high-resolution computed tomography, with contiguous axial and coronal sections of 1-mm thickness, was obtained to investigate the structure of the inner ear [32–34].

### Genetic examination

Dried blood spot specimens were collected from the patients and their family members, and genomic DNA was extracted using a MagCore HF16 Automatic DNA/RNA Purification system (RBC Bioscience Corp., Taiwan) with a MagCore Genomic DNA Tissue Kit (RBC Bioscience Corp., Taiwan) according to the manufacturer's instructions [35, 36]. We standardized a genetic examination protocol for mutation screening of three common deafness genes: *GJB2*, *SLC26A4*, and *MT-RNR1* [37, 38] in all patients. Sanger sequencing was performed on both exons of *GJB2*. Real-time PCR was performed on two mutation hotspots of *SLC26A4* (c.919-2A>G and p.H723R), and on the m.1555A>G mutation of *MT-RNR1*. Patients with enlarged vestibular aqueduct (EVA), a common inner ear malformation caused by recessive *SLC26A4* mutations [39, 40], were further subjected to a next-generation sequencing (NGS)-based diagnostic panel targeting all the exons of *SLC26A4*. The NGS data were filtered and analyzed as previously described [41]. The Deafness Variation Database (http://deafnessvariationdatabase.org/) and ClinVar (https://www.ncbi.nlm.nih.gov/clinvar/) were used to identify known causative variants. All subjects and/or their parents provided informed consent before genetic testing, and all procedures were approved by the Research Ethics Committees of National Taiwan University Hospital, National Center for Maternal and Child Health of Mongolia, and the EMJJ Otolaryngology Hospital of Mongolia.

### Haplotype analyses

**Fig 1:**
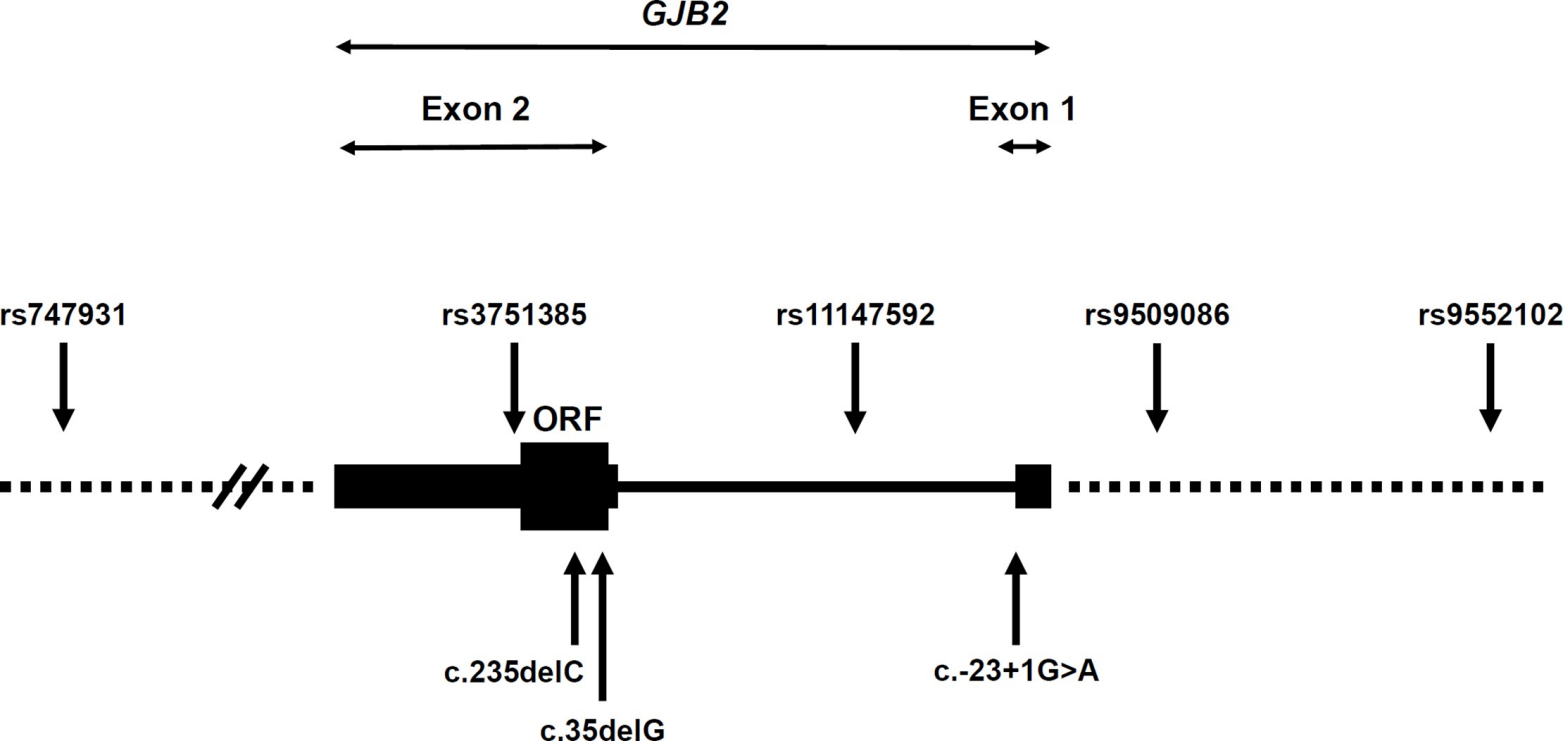
Positions of the single-nucleotide polymorphisms (SNPs) we genotyped, relative to the *GJB2* gene. The *GJB2* gene consists of two exons (coding exon, thick black box; untranslated regions, thin black boxes; intron, thin line). The relative positions of the five SNPs (rs747931, rs3751385, rs11147592, rs9509086, and rs9552102) and the three *GJB2* mutations (c.235delC, c.35delG, and c.-23+1G>A) are shown by arrows.

Five single-nucleotide polymorphisms (SNPs) within or in the vicinity of *GJB2*, namely rs747931, rs3751385, rs11147592, rs9509086, and rs9552102, were selected and genotyped by Sanger sequencing (Fig 1). Haplotypes were constructed with these five SNP markers in the mutant alleles from patients with *GJB2*: c.-23+1G>A, c.35delG, or c.235delC mutations, and compared to those in the wild-type alleles from 47 Mongolian controls with normal hearing. To investigate genetic relationships with other populations, we further selected 14 c.235delC homozygotes, comprising 11 Han Taiwanese and three Han Chinese from our cohort [36, 37], and determined their haplotypes. Meanwhile, population-specific and mutation-specific haplotype structures were also generated from the data of the 1000 Genomes Project using the LDlink web-based tool [42].

### Statistical analyses

Differences between groups were tested with Fisher’s exact test (SPSS 22.0 software, IBM SPSS, Armonk, NY). Corresponding *p* values < 0.05 were interpreted as being statistically significant.

## Results

In the 188 unrelated Mongolian families with SNHI, nine different mutant *GJB2* alleles and three mutant *SLC26A4* alleles were identified (Table 1). The allele frequency of *GJB2* mutations (10.6%; 40/376) was higher than that of *SLC26A4* mutations (2.4%; 9/376) and that of the mitochondrial m.1555A>G mutation (1.1%; 2/188). More prevalent *GJB2* mutations included c.-23+1G>A (allele frequency = 3.2%; 12/376), c.235delC (2.1%; 8/376), and c.35delG (1.6%; 6/376); whereas the most prevalent *SLC26A4* mutation was c.919-2A>G (2.1%). The *SLC26A4* c.2168A>G mutation, common in the East Asian populations [19–21], was not detected in Mongolian patients in this study. Except for *GJB2* c.235delC, none of these variants was identified in the 47 normal-hearing controls. Because of the limited number of the normal-hearing controls, there was no difference in the allele frequencies of these variants between the patient and control groups (Fisher’s exact test, all *p* > 0.05). However, as all these variants have been previously shown to be disease-causing in other populations, they were interpreted as causative pathogenic mutations in this study.

**Table 1:** Mutant alleles detected in the 188 deaf families and 47 normal-hearing controls.

Nucleotide change	Amino acid change	Allele no. in patients (%)^#^	Allele no. in controls (%)^†^
*GJB2*			
c.-23+1G>A	NA	12 (3.2)	0 (0)
c.235delC	p.Leu79Cysfs*3	8 (2.1)	1 (1.1)
c.35delG	p.Gly12Valfs*2	6 (1.6)	0 (0)
c.109G>A	p.Val37Ile	4 (1.0)	0 (0)
c.299_300delAT	p.His100Argfs*14	4 (1.0)	0 (0)
c.35dupG	p.Val13Cysfs*35	2 (0.5)	0 (0)
c.560_605dup	p.Cys202*	2 (0.5)	0 (0)
c.269T>C	p.Leu90Pro	1 (0.3)	0 (0)
c.508_511dupAACG	p.Ala171Glufs*40	1 (0.3)	0 (0)
Total		40 (10.6)^K^	1 (1.1)^K^
*SLC26A4*			
c.919-2A>G	NA	7 (2.1)	0 (0)
c.281C>T	p.Thr94Ile	1 (0.3)	0 (0)
c.2027T>A	p.Leu676Gln	1 (0.3)	0 (0)
Total		9 (2.4)	0 (0)
*MT-RNR1*			
m.1555A>G	NA	2 (1.1)	0 (0)

^#^ 376 *GJB2*, 376 *SLC26A4*, and 188 *MT-RNR1* alleles.

^†^ 94 *GJB2*, 94 *SLC26A4*, and 47 *MT-RNR1* alleles.

^K^ *p* < 0.01 by Fisher’s exact test.

NA, Not available.

**Fig 2:**
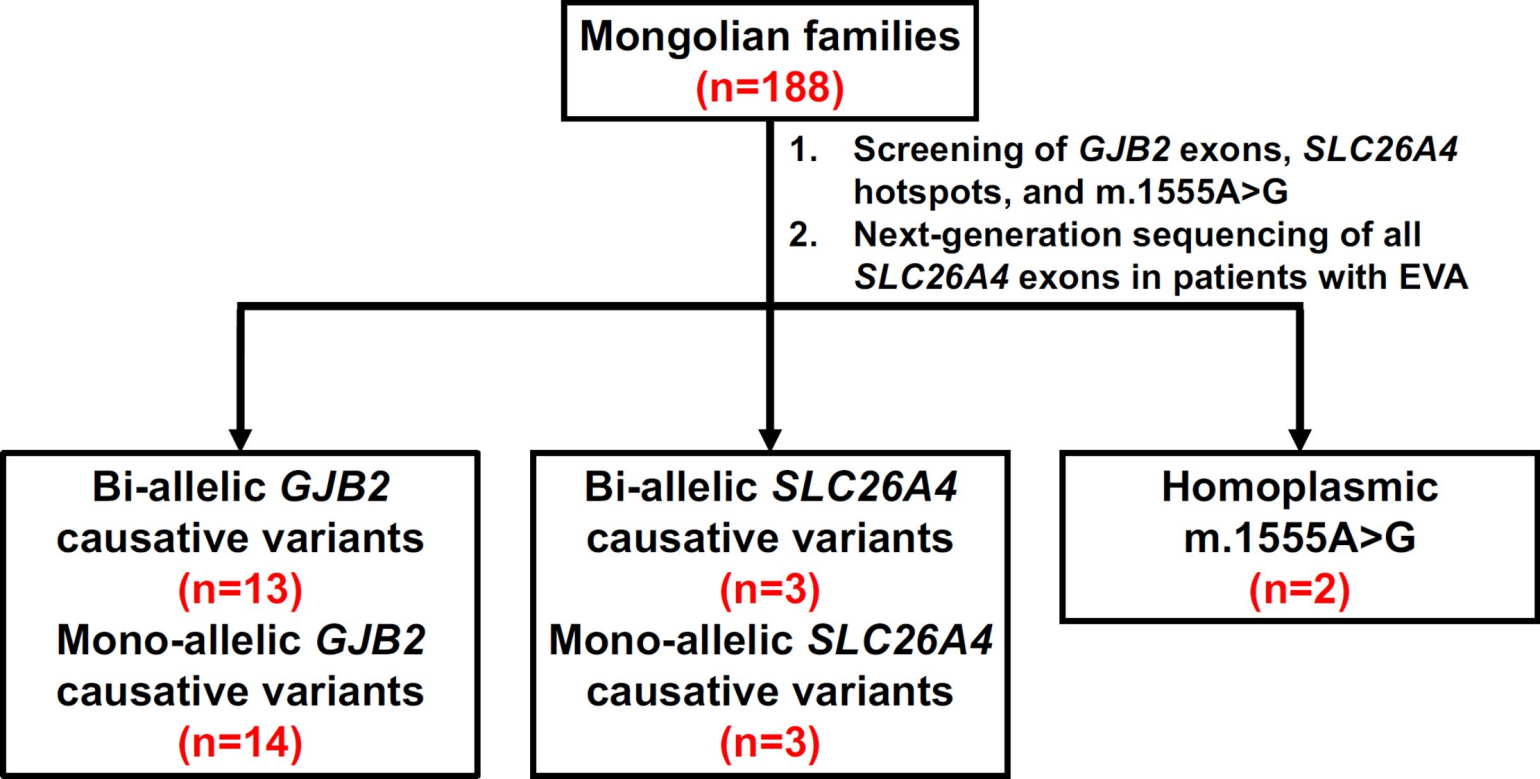
Summary of genetic results in the 188 Mongolian families with sensorineural hearing impairment. Bi-allelic and mono-allelic *GJB2* mutations were identified in 13 and 14 families, respectively. Bi-allelic and mono-allelic *SLC26A4* mutations were identified in three and three families, respectively. Homoplasmic m.1555A>G mutation was detected in two families. EVA, enlarged vestibular aqueduct.

Definite genetic diagnosis was achieved in 18 of the 188 families (9.6%), including 13 with bi-allelic *GJB2* mutations, three with bi-allelic *SLC26A4* mutations, and two with homoplasmic m.1555A>G mutations (Table 2 & Fig 2). In addition, mono-allelic *GJB2* and *SLC26A4* mutations were detected in the probands of 14 and three families, respectively. All the probands in the three families with mono-allelic *SLC26A4* mutations showed EVA on imaging studies. According to previous reports [43–46], a second occult mutant *SLC26A4* allele, which could not be detected using current sequencing techniques, might exist in these three families, and presumably SNHI in the affected members could be attributed to *SLC26A4* mutations.

**Table 2:** Genetic results of the 188 Mongolian families.

Genes	Variants	Numbers	Percentage (%)
*GJB2*	Bi-allelic		
	c.-23+1G>A/c.235delC	3	1.6
	c.-23+1G>A/c.35delG	2	1.0
	c.-23+1G>A/c.299_300delAT	2	1.0
	c.35delG/c.35dupG	1	0.5
	c.-23+1G>A/c.269T>C	1	0.5
	c.-23+1G>A/c.559_604dup	1	0.5
	c.235delC/c.235delC	1	0.5
	c.235delC/c.299_300delAT	1	0.5
	c.235delC/c.559_604dup	1	0.5
	Mono-allelic		
	c.109G>A/WT	4	2.1
	c.-23+1G>A/WT	3	1.6
	c.35delG/WT	3	1.6
	c.35dupG/WT	1	0.5
	c.235delC/WT	1	1.0
	c.299_300delAT/WT	1	0.5
	c.508_511dupAACG/WT	1	0.5
*SLC26A4*	Bi-allelic		
	c.919-2A>G/c.919-2A>G	1	0.5
	c.919-2A>G/c.281C>T	1	0.5
	c.919-2A>G/c.2027T>A	1	0.5
	Mono-allelic		
	c.919-2A>G/WT	3	1.6
*MT-RNR1*	m.1555A>G	2	1.1

WT, wild-type.

To investigate whether prevalent mutations in the Mongolian patients derived from common origins, we performed haplotype analyses by genotyping five SNPs, in the patients and in 47 normal-hearing Mongolian controls (Table 3). Our results revealed that all six chromosomes with the *GJB2* c.35delG mutation segregated exclusively with the A-A-C-G-T haplotype (Fisher’s exact test, *p* = 0.002; compared to the 94 control chromosomes), and all eight chromosomes with the *GJB2* c.235delC mutation segregated exclusively with the A-G-T-T-A haplotype (Fisher’s exact test, *p* < 0.001; compared to the 94 control chromosomes). On the other hand, the *GJB2* c.-23+1G>A mutation was associated with a major haplotype G-G-C-T-A (9/12 chromosomes) and a minor haplotype A-G-C-T-A (3/12 chromosomes), which also differed in haplotype distribution as compared to the control chromosomes (Fisher’s exact test, *p* < 0.001). These results indicated founder effects for all the three prevalent *GJB2* mutations in the Mongolian patients.

**Table 3:** Haplotype analyses of *GJB2* alleles in the Mongolian patients.

Haplotype	c.-23+1G>A	c.35delG	c.235delC	wild-type
A-A-C-G-T	0	6*	0	31
A-G-C-T-A	3	0	0	27
A-G-T-T-A	0	0	8*	19
G-A-C-G-T	0	0	0	7
G-G-C-T-A	9*	0	0	6
A-A-C-G-A	0	0	0	3
A-A-C-T-A	0	0	0	1
Total	12	6	8	94

Differences between mutant and wild-type alleles for each haplotype were tested with Fisher’s exact test.

*p < 0.05

Population-specific haplotype structures, generated from the data of the 1000 Genomes Project, revealed that the Mongolian population shares a closer genetic background with East Asians than with the Europeans and South Asians (S1 Table). The three predominant *GJB2* haplotypes, namely A-A-C-G-T, A-G-C-T-A, and A-G-T-T-A, were the same in the Mongolian and East Asian populations; whereas a common *GJB2* haplotype in the European and South Asian populations, G-G-C-T-A, was relatively rare in the Mongolians and East Asians.

However, when focused on mutation-specific haplotype structures, the *GJB2* c.235delC mutation in Mongolians originated from a common ancestor with other East Asians, whereas the *GJB2* c.35delG originated from a common ancestor with the Europeans (Table 4). Of the 14 c.235delC homozygotes selected from our cohort, 21 of the 22 Taiwanese chromosomes and five of the six Chinese chromosomes with *GJB2* c.235delC shared the same A-G-T-T-A haplotype with the 8 Mongolian chromosomes with c.235delC. An additional eight chromosomes with the c.235delC mutation were identified from East Asians in the 1000 Genomes Project database, and all were determined by LDlink to segregate the A-G-T-T-A haplotype as well. Both lines of evidence indicated a common founder for *GJB2* c.235delC in Mongolians and that in East Asians. Similarly, in the 1000 Genomes Project database, nine and two chromosomes with the c.35delG mutation were identified from the Europeans and Admixed Americans, respectively; and 10 of the 11 chromosomes shared the same A-A-C-G-T haplotype with the 6 Mongolian chromosomes with c.35delG, indicating a common founder for *GJB2* c.35delG in the Mongolians and Europeans. Unfortunately, the haplotype structures with the *GJB2* c.-23+1G>A mutation could not be determined from the 1000 Genomes Project database to investigate the common ancestry of this mutation in Mongolians and South Asians.

**Table 4:** Mutation-specific haplotype structures of *GJB2* c.235delC and c. 35delG in different populations.

**Haplotype**	**Mongolians**	**Taiwanese**	**Chinese**	**East Asians**
c. 235delC				
A-G-T-T-A	8	21	5	8*
G-G-T-T-A	0	1	1	0*
**Haplotype**	**Mongolians**	**Europeans**	**Admixed Americans**	
c. 35delG				
A-A-C-G-T	6	8*	2*	
G-A-C-G-T	0	1*	0*	

* The haplotype structures were determined from the data of the 1000 Genomes Project using the LDlink tool.

## Discussion

Our results unraveled a unique genetic profile in Mongolian patients with SNHI as compared to other European and Asian populations. Notably, three *GJB2* mutations that are prevalent in other populations, including c.35delG in Caucasians [5–7], c.235delC in East Asians [10–12], and c.-23+1G>A in Southwest and South Asians [13–16], were simultaneously detected in Mongolian patients. To our knowledge, this is the first study in the literature to identify these three common *GJB2* mutations with significant allele frequencies in a single ethnic group.

Haplotype analyses further confirmed founder effects for each of the three mutations, indicating that each mutation derived from its individual ancestral origin independently. It was reported that the c.35delG mutation stemmed from the Volgo-Ural region of Central Asia approximately 11,800 years ago [47] and then spread throughout Europe along the two Neolithic population transportation routes [48]. The c.235delC mutation arose near the Baikal Lake or the Altai-Sayan region approximately 11,500 years ago, and then spread into East Asia [49–51]. The c.-23+1G>A mutation was estimated to occur approximately 800 years ago, and spread into Mongolia, Siberia, or South Asia with the Turkic migration in the 13th–14th centuries [52]. The concurrence of the c.35delG, c.235delC, and c.-23+1G>A mutations in the Mongolian patients might reflect the geographic location of Mongolia as a crossroads in this migration. In addition, the territorial expansion of the Mongol Empire in the 13th century might also have enhanced gene flow between Mongolians and other populations who lived in Europe, Central Asia, East Asia, and the Indian subcontinent [30].

Prior to the current study, several studies have investigated the genetics of SNHI in Mongolian patients (Table 5). Most of these studies were performed on patients recruited from the Inner Mongolia region of China or northwest China, and included patients of non-Mongolian ethnicity. Dai et al. sequenced coding exons of *SLC26A4* in 135 patients from Inner Mongolia, and found that 12.6% (17/135) patients carried bi-allelic *SLC26A4* mutations. However, only 31 of the 135 patients were Mongolians, and the authors did not classify their genetic results by ethnicity [23]. Yang et al. screened the coding regions of *GJB2*, *SLC26A4*, and *MT-RNR1* in 189 deaf patients from northwest China, of whom only 19 were Mongolians. The authors did not identify any *GJB2* mutations, and reported the *SLC26A4* c.919-2A>G mutation as the most common deafness mutation in the Mongolian patients [24]. Liu et al. screened nine common mutations of *GJB2*, *SLC26A4*, *MT-RNR1*, and *GJB3* in 738 deaf children recruited from the Inner Mongolia region of China, including 216 Mongolians. The authors also reported a higher prevalence of *SLC26A4* mutations than that of *GJB2* mutations [25]. In contrast to these previous reports, our study showed that *GJB2* mutations are more prevalent than *SLC26A4* mutations in Mongolian patients with SNHI. This difference between our study and previous reports might result from the more thorough sequencing strategy we adopted, since the *GJB2* c.-23+1G>A mutation, which is prevalent in Mongolians but rare in other East Asian populations, was not targeted in previous reports. It is also notable that the genetic structure of the Mongolian people who live in China could have been influenced by that of other races through inter-population marriage.

**Table 5:** Summary of previous studies and our study on the genetic results of Mongolian patients.

Reference	Patients	Target regions	**Main results**
Dai *et al*. [23]	135 patients from the Inner Mongolia region of China, including 94 Han Chinese, 31 Mongolians, 7 Manchurians, and three Hui	The coding exons of *SLC26A4*	12.6% (17/135) patients carried bi-allelic *SLC26A4* mutations. The most common mutation was c.919-2A>G. Mutations in the Mongolian patients were not specified.
Tekin *et al*. [26]	534 Mongolian patients from Mongolia	The coding exon (exon 2) of *GJB2* and the c.23+1G>A mutation in intron 1	24 (4.5%) and 29 (5.4%) patients carried bi- and mono-allelic *GJB2* mutations, respectively. The most common mutations were *GJB2* c.23+1G>A (3.5%) and c.235delC (1.5%).
Yang *et al*. [24]	189 patients from the northwest of China, including 121 Tibetans, 49 Tu, and 19 Mongolians	The coding regions of *GJB2*, *SLC26A4*, and *MT-RNR1*	The most common mutation in the Mongolian patients was *SLC26A4* c.919-2A>G. No *GJB2* mutations were detected in the Mongolian patients, one of whom was found to carry the *MT-RNR1* m.1555A>G mutation.
Liu *et al*. [25]	738 patients from the Inner Mongolia region of China, including 486 Han Chinese, 216 Mongolians, 24 Manchurians, 6 Hui, and 6 Daur	Nine common mutations in four deafness genes, including *GJB2*, *SLC26A4*, *GJB3*, and *MT-RNR1*	Among the 216 Mongolian patients, 36 had *GJB2* mutations and 42 had *SLC26A4* mutations.
This study	188 unrelated Mongolian patients from Mongolia	All exons of *GJB2* and *SLC26A4*, and the coding region of *MT-RNR1*	Definite genetic diagnosis was achieved in 18 (9.6%) of the 188 patients, including 13 with bi-allelic *GJB2* mutations, 3 with bi-allelic *SLC26A4* mutations, and two with homoplasmic *MT-RNR1* m.1555A>G mutation.

In the present study, definite genetic diagnoses could be achieved in 18 (9.6%) of the 188 families, comprising 13 (6.9%) with bi-allelic *GJB2* mutations, three (1.6%) with bi-allelic *SLC26A4* mutations, and two (1.1%) with homoplasmic m.1555A>G mutations, by screening the three common deafness genes. We additionally identified mono-allelic *GJB2* or *SLC26A4* mutations in 17 families (9.0%); however, given the recessive inheritance pattern of mutations in these two genes, the genetic results in these families were regarded as unconfirmed. Accordingly, the rate of confirmed results in our study is lower than that documented in other studies on European [53, 54] and Asian [37, 55–57] populations. This low rate is consistent with the results of the study by Tekin et al., who reported a low rate of 4.5% with bi-allelic *GJB2* mutations in Mongolian patients from a deaf school in Ulaanbaatar [26]. Liu et al. detected *GJB2* mutations in 36 (16.6%) of their 216 Mongolian patients from the Inner Mongolia region of China [25]. However, these comprised patients with both bi-allelic and mono-allelic mutations, and the actual percentage of patients with confirmed *GJB2* mutations might be considerably lower than 16.6% [25].

The relatively low mutation rates of deafness genes in Mongolians have been ascribed to lower assortative mating rate and decreased genetic fitness of the deaf in Mongolia as compared to other populations [26]. It has been suggested that the introduction of sign language, establishment of residential schools for the deaf, and appearance of intense assortative mating among the deaf might have relaxed the genetic selection against deafness and contributed to high frequency of *GJB2* deafness in many western populations [58]. Indeed, low frequency of < 10% of deafness-associated *GJB2* mutations has been reported in several populations with lower socioeconomic status, such as patients in Sudan [59], Kenya [59], Indonesia [60], and Cameroon [61].

Notably, two hearing-impaired patients in this study were identified to have the mitochondrial m.1555A>G mutation. Their medical history revealed that both patients were exposed to aminoglycosides. According to a recent report, the Asia-Pacific region is the largest market for the aminoglycoside industry, probably owing to a high incidence rate of tuberculosis (http://www.grandviewresearch.com/industry-analysis/aminoglycoside-market). To prevent aminoglycoside-induced hearing loss, it might be reasonable to perform mutational screening of the *MT-RNR1* gene before the initiation of antibiotic therapy, especially in regions where aminoglycosides are frequently used [62, 63].

By comprehensively sequencing both *GJB2* exons, we identified bi-allelic and mono-allelic recessive *GJB2* mutations in 13 (6.9%) and 14 (7.4%) of the 188 Mongolian families, respectively. This finding is also consistent with Tekin et al., who reported mono-allelic *GJB2* mutations in 5.4% of their Mongolian patients [26]. Patients with mono-allelic recessive *GJB2* mutation might have occult mutations in the non-coding regions of *GJB2*, such as untranslated exon 1, intron 1, promoter, enhancer, or other regulatory elements, leading to compound heterozygosity, which has been observed previously for *GJB2* mutations [64, 65]. Alternatively, mutations in other gap junction genes might modulate the pathogenicity of *GJB2* mutations and contribute to hearing impairment via digenic or polygenic inheritance [66–68]. The third possibility is that, instead of *GJB2* mutations, hearing impairment is in fact caused by mutations in other deafness genes, and these “mono-allelic” patients are incidental carriers of certain *GJB2* variants that are prevalent in the population [69].

The major strength of this study lies in the demonstration of the mutation spectra of all the three common HHI genes in a single large cohort of pure Mongolian ethnicity. However, some limitations of this study merit further discussion. Genetic mutations, with confirmed and unconfirmed results counted together, were only identified in 35 (18.6%) of the 188 families; for the other families, the etiology remained unclear. It is conceivable that in a certain portion of these families SNHI may be caused by mutations in other deafness genes, especially in families with multiple affected members. In addition, SNHI of acquired causes, such as perinatal insults or congenital cytomegalovirus (cCMV) infection, may be more prevalent in developing countries like Mongolia than in industrialized countries [70]. Recently, NGS technology, which enables the sequencing of a large number of genes simultaneously, has proven to be a powerful tool for addressing the genetically heterogeneous disorder HHI [38, 71, 72]. We are currently using an NGS-based diagnostic panel to analyze the genetic etiology in the undiagnosed families of our Mongolian cohort [38, 41], and have now identified causative *MYO15A* mutations in a multiplex family. The employment of NGS-based genetic examination and cCMV screening may help improve the evaluation, diagnosis, and management of Mongolian patients with SNHI.

In conclusion, by performing comprehensive genetic examination and haplotype analyses in a large Mongolian cohort with SNHI, this study throws light on the genetic epidemiology of HHI in Mongolians. It also provides insights into the development of the unique mutation spectra observed in Mongolians. These findings may have clinical implications for the refinement of molecular diagnostics in Mongolian patients, as well as scientific implications for the delineation of genetic relationships among the Eurasian populations.

## Supporting information

S1 Table Haplotype distributions of *GJB2* in different populations. (DOCX)
