# Supplementary material for: Unique spectra of deafness-associated mutations in Mongolians provide insights into the genetic relationships among Eurasian populations
Source: PLoS One. 2018 Dec 21;13(12):e0209797. doi: 10.1371/journal.pone.0209797 (PMC6303056; doi:10.1371/journal.pone.0209797)
Supplement: S1 Table — (DOCX) [file pone.0209797.s001.docx]

| **Haplotype** | **Mongolians** | **East Asians** | **Europeans** | **South Asians** |
| --- | --- | --- | --- | --- |
| A-A-C-G-T | 31 (33%) | 335 (33%) | 41 (4%) | 41 (4%) |
| A-G-C-T-A | 27 (29%) | 326 (32%) | 319 (32%) | 389 (40%) |
| A-G-T-T-A | 19 (20%) | 191 (19%) | 167 (17%) | 179 (18%) |
| G-A-C-G-T | 7 (7%) | 42 (4%) | 20 (2%) | 19 (2%) |
| G-G-C-T-A | 6 (6%) | 50 (5%) | 190 (19%) | 171 (17%) |
| A-A-C-G-A | 3 (3%) | 12 (1%) | 78 (8%) | 44 (4%) |
| A-A-C-T-A | 1 (1%) | 1 (0%) | 0 (0%) | 1 (0%) |
| Others | 0 (0%) | 51 (5%) | 191 (19%) | 134 (14%) |
| Total | 94 | 1008 | 1006 | 978 |

The value in brackets represents the percentage of the haplotype in each population
